# AAV2 and AAV9 tropism and transgene expression in the mouse eye and major tissues after intravitreal and subretinal delivery

**DOI:** 10.3389/fddev.2023.1148795

**Published:** 2023-07-12

**Authors:** Sanna Koponen, Emmi Kokki, Toni Tamminen, Seppo Ylä-Herttuala

**Affiliations:** ^1^ Molecular Medicine Group, A. I. Virtanen Institute for Molecular Sciences, University of Eastern Finland, Kuopio, Finland; ^2^ Department of Ophthalmology, Institute of Clinical Medicine, University of Eastern Finland, Kuopio, Finland; ^3^ Gene Therapy Unit, Kuopio University Hospital, Kuopio, Finland

**Keywords:** AAV, gene therapy, biodistribution, tropism, intravitreal injection, subretinal injection

## Abstract

**Introduction:** The eye is an excellent target for gene therapy because of its anatomical features. Gene therapy to treat ocular disorders relies on efficient gene delivery and transgene expression in the target cells. The aim of this study was to compare the biodistribution and safety of two different AAV serotypes after intravitreal (IVT) and subretinal injections.

**Methods:** AAV2 (1 × 10^12^ vg/mL) and AAV9 (5 × 10^12^ vg/mL) vectors expressing an enhanced green fluorescent protein (EGFP) and an AAV9-empty (6 × 10^11^ vg/mL) vector were injected intravitreally or subretinally into both eyes of adult C57Bl/OlaHsd mice. The biodistribution of the viral vectors in the eye and off-target tissues was studied using qPCR. GFP expression was studied from cryosections, and GFP transduction efficacy was verified using immunohistostaining for GFP. In addition, electroretinography (ERG) was used to assess the effect of vectors on retinal function.

**Results:** In addition to the eyes, viral vector copies were found in distant off-target tissues such as the liver, especially after AAV9-EGFP IVT and subretinal injections. AAV9-EGFP injections showed more GFP expression throughout the retina compared to AAV2-EGFP. AAV2-EGFP IVT showed transgene expression mainly in the ganglion cell layer, whereas subretinal injection showed GFP expression in the retinal pigment epithelium. In addition, GFP was expressed at a moderate level in the liver after both injection routes of AAV9 and in parts of the brain after all injection groups except AAV9-empty. Lowered a- and b-amplitude values were seen in ERG in both scotopic and photopic experiments after AAV9-EGFP subretinal injection compared to all other groups.

**Discussion:** This study shows that intraocular injection of AAV2 and AAV9 transduces retinal cells. Although the more efficient transduction of the retina, negative effect on the retinal function, and off-target transgene expression of AAV9 makes AAV2 a more suitable gene delivery vector to treat ocular disorders.

## 1 Introduction

Due to static, dynamic, and metabolic barriers, delivery of drug and gene therapy to the back of the eye is challenging (Gaudana et al., 2010). The blood–retinal barrier limits the penetration of almost all drugs from the systemic circulation to the eye. Topical delivery to the back of the eye is restricted by static barriers to penetration including the conjunctiva, sclera, choroid, retinal pigment epithelium, and cornea (Rowe-Rendleman et al., 2014). Although invasive, IVT injection remains the most common delivery method of drugs and gene therapy to the posterior segment (Grzybowski et al., 2018). Intraocular injections achieve the highest intraocular bioavailability by bypassing several anatomic and dynamic barriers at the back of the eye (Rowe-Rendleman et al., 2014).

Injection to vitreous humor possesses advantages like relatively low cost and level of familiarity to ophthalmologists, whereas subretinal injection is a more demanding procedure (Koponen et al., 2021). In patients, IVT injection is administered through the sclera into the vitreous cavity under local analgesia, whereas subretinal injection is a more invasive method that requires surgery (Ochakovski et al., 2017). A subretinal injection can be administered by the pars plana vitrectomy approach (Davis et al., 2019).

The vitreous may decrease the efficacy of gene delivery in various ways (Peeters et al., 2005). Drugs are eliminated from the vitreous due to dynamic clearance mechanisms, such as anterior bulk aqueous- or posterior vitreoretinal-choroidal flow (Rowe-Rendleman et al., 2014). As a result, repeated injections are needed to maintain therapeutic concentrations. In chronic ocular disease, frequent injections pose a burden for both the patient and the healthcare system and increase the risk of injection-related adverse events, including intraocular inflammation, hemorrhage, ocular hypertension, and cataract, although the overall risk of a severe adverse event is low (Jager et al., 2004). The risk of intraocular inflammation is found to be low after IVT injections (Li et al., 2021), and safer and easier techniques for intraocular injections have been developed (Watanabe et al., 2018). Thus, intraocular gene therapy with sustained long-term expression offers a promising alternative to traditional drug treatments.

The eye is an excellent target for gene therapy because of its anatomical features (Petit et al., 2016). The eye is relatively immune-privileged and easy to access, and blood–ocular barriers limit systemic biodistribution and side effects of the introduced genetic material (Moore et al., 2017). Thus, it is widely clinically studied in hereditary retinal diseases, including Leber hereditary optic neuropathy (NCT04912843, 2022), X-linked retinitis pigmentosa (NCT04312672, 2022), and choroideremia (NCT04483440, 2023) and as well as in common acquired ocular disorders like age-related macular degeneration (NCT05407636, 2023) and diabetic retinopathy (NCT04567550, 2023). Ophthalmological diseases are the third most common indication in clinical trials (Zhao et al., 2022). Several clinical trials using AAV as a gene delivery vector for ophthalmic disorders have been conducted or are still ongoing (Ghoraba et al., 2022; Tian et al., 2022). In addition, the AAV2-mediated gene therapy Luxturna (voretigene neparvovec-rzyl) to treat inherited retinal dystrophy was approved in 2017 by the US FDA (FDA, 2022).

Successful gene therapy relies on safe and efficient gene delivery into the target cells. Although gene transfer of nonviral vectors by physical or chemical methods enables repeated administration, a low transduction efficiency limits their clinical use. Plasmid DNA and small DNA and RNA molecules are nonpathogenic and unlikely to be immunogenic and mutagenic (Bordet and Behar-Cohen, 2019; Butt et al., 2022). Electroporation of plasmid DNA into the ciliary muscle has been studied in patients with noninfectious uveitis (NCT03308045, 2022). However, the production of therapeutic levels of secreted proteins in the posterior segments of the eye remains preclinical (Touchard et al., 2018).

Due to their superior transduction efficacy, viral vectors have been the most frequently used gene delivery method in all gene therapy trials (Arabi et al., 2022), including ocular gene therapy (Koponen et al., 2021). Especially, the use of adeno-associated virus (AAV) vectors has become more popular, now constituting almost half of the vectors in clinical trials (Zhao et al., 2022). AAV is a nonenveloped virus containing a single-stranded DNA genome (Naso et al., 2017). AAVs have several features that make them efficient gene delivery vectors. AAV vectors have a broad tropism in different tissues; their use results in long-term expression of the transgene, and they have been shown to be less immunogenic than several other viral vectors (Zincarelli et al., 2008; Li and Samulski, 2020). Despite the advantages and promising results from preclinical and clinical trials, AAVs have been shown to elicit immune responses (MacLachlan et al., 2011; Russell et al., 2017), and these responses can limit efficient gene transduction (Korpela et al., 2022). Innate and adaptive immune responses are dose-dependent after IVT (Cukras et al., 2018) and subretinal injections in preclinical (Barker et al., 2009) and clinical trials (Le Meur et al., 2018). However, in these studies, inflammatory responses were mild, transient, and, in clinical trials, controlled by local or systemic steroids. Although both intraocular routes have been shown to elicit immune responses, subretinal injections are considered to be less immunogenic than IVT injections (Li et al., 2008). In addition, subretinal injection has not had an effect on repeated administration in the other eye, whereas repeated IVT administration to the other eye resulted in humoral immune response blocking the vector expression. Pre-existing antibodies can also reduce the efficacy of gene therapy. Antibodies against different AAV serotypes have been observed in human populations (Calcedo et al., 2009; Boutin et al., 2010). However, in the vitreous humor, the level of pre-existing antibodies against AAVs is lower than the levels in the serum (Lee et al., 2019). The condition of the blood–retinal barrier can affect the levels of neutralizing antibodies in the vitreous. In one clinical trial with patients who presented with pre-existing antibodies against AAV2, subretinal injection of AAV2-mediated gene therapy caused only four of 15 subjects to experience a significant increase of circulating anti-AAV2 antibodies in serum, suggesting a limited systemic response (Jacobson et al., 2012).

Few studies have reported assessing the biodistribution of viral vectors and transgene expression after IVT or subretinal injection of AAV-based vectors. The aim of this study was to compare the gene expression, biodistribution, and safety of two different AAV serotypes, AAV2 and AAV9, after IVT and subretinal injection into mouse eyes.

## 2 Materials and methods

### 2.1 Production of AAV vectors

AAV serotypes 2 and 9 expressing enhanced green fluorescent protein (EGFP) with the cytomegalovirus (CMV) promoter and woodchuck posttranscriptional regulatory element (WPRE) and AAV9-empty were used in the study. Vectors were produced as described earlier by Zolotukhin et al., 1999 with a modified calcium phosphate precipitation protocol. Low-passage HEK-293T cells (human embryonic kidney 293 cells with SV40 large T-antigen) were cultured in Dulbecco’s modified Eagle's medium (DMEM) supplied with 10% fetal calf serum (FBS), penicillin, and streptomycin; 85%–95% confluent cells were transfected with calcium phosphate precipitation. Cells were co-transfected with vector plasmid, helper plasmids pDG (kindly provided by Dr. Jurgen Kleinschmidt, DKFZ, Heidelberg, Germany), and pXX6-80 and packaging plasmid (pAAV2/2, pAAV2/9). After 24 h of the transfection, the medium was replaced with fresh DMEM +10% FBS + penicillin/streptomycin, and after 48 h, cells were harvested. Three freeze–thaw cycles in lysis buffer (0.15 M NaCl, 50 mM Tris-HCl; pH 8.5) were used to release AAV vectors from cells. The vector-containing media was purified by iodixanol-gradient centrifugation and with the Amicon purification method. Purified fractions were sterile filtered with PBS through a 0.2-µm filter and stored at −70°C. Viral vector titers for AAV2-EGFP (1.7 × 10^13^ vg/mL), AAV9-EGFP (1.2 × 10^13^ vg/mL), and AAV9-empty (4.1 × 10^13^ vg/mL) were determined with qPCR, as described in Suoranta et al., (2021). Vectors were further diluted in 1x Dulbecco’s phosphate-buffered saline (DPBS) before injections to titers 1 × 10^12^ vg/mL for AAV2-EGFP, 5 × 10^12^ vg/mL for AAV9-EGFP, and 6 × 10^11^ vg/mL for AAV9-empty.

### 2.2 *In vivo* studies

Thirty-one male C57BL/6JOlaHsd mice (Envigo, United States) aged 14–19 weeks were used in the study. The mice were housed in a controlled environment in a conventional animal facility with a 12-h light/dark cycle and had access to food (Teklad 2016S, Envigo, United States) and water *ad libitum*. All animal procedures were approved by the Animal Experiment Board in Finland and carried out according to the guidelines of the Experimental Animal Committee of the University of Eastern Finland.

Intraocular injections were made either IVT or subretinally. Animals were anesthetized with a mixture of ketamine (Ketaminol vet 50 mg/mL, Intervet International B.V., Netherlands) and medetomidine hydrochloride (Domitor vet 1 mg/mL, Orion Pharma, Finland), and pupils were dilated with tropicamide (Oftan tropicamide 5 mg/mL, Santen Oy, Finland). Topical analgesia was given prior to injections with oxybuprocaine (Oftan Obucain, 4 mg/mL, Santen Oy, Finland) eye drops. Eyes were hydrated with carbomer eye gel during the procedure (Viscotears 2 mg/g, Dr. Gerhard Mann chem.-pharm. Fabrik GmbH, Germany). Injections were performed with a 34-gauge needle and a 10-µL microsyringe (Hamilton Bonaduz AG, Bonaduz, Switzerland). IVT injection was performed through the sclera about 2 mm to the superior margin of the limbus, and subretinal injection was performed via a transscleral route through the choroid and Bruch’s membrane without penetrating the retina. The injection was made slowly, and after the injection, the needle was held in place for half a minute to prevent leakage of the vector. Both eyes received 2 µL of viral vectors AAV2-EGFP (1 × 10^12^ vg/mL), AAV9-EGFP (5 × 10^12^ vg/mL), and AAV9-empty (6 × 10^11^ vg/mL), and after the injections, the injection site was inspected for bleeding under a microscope. Eyes with bleeding or unsuccessful injection were discarded from the study. Anesthesia was reversed by atipamezole (Antisedan, vet 5 mg/mL, Orion Pharma, Finland).

Mice were euthanized 1 month after the IVT or subretinal injection with carbon dioxide and perfused with DPBS transcardially. Samples from the eyes, brain, lung, heart, liver, spleen, kidneys, testicles, lymph nodes, and quadriceps muscles were harvested for qPCR and histology (Table 1). Samples for qPCR were snap-frozen in liquid nitrogen and stored at −70°C. All tissue samples for histology were fixed with 4% paraformaldehyde (PFA) in DPBS (pH 7.4) for 20–24 h except eyes, which were fixed for 4 h. After the fixation, all samples except eyes were transferred to 15% sucrose in milli-Q water for 48 h. Eyes were transferred to 20% sucrose in milli-Q water. For frozen sections, optimal cutting temperature compound (OCT) embedded eyes were treated after the fixation with 15% sucrose for 30 min, 20% sucrose for 4 h, and 30% sucrose for 48 h. The samples were embedded in an OCT (Sakura Finetek, Europe BV, Netherlands) or paraffin. Paraffin blocks were sectioned at 5 μm using a Microm HM 3555s (Thermo Scientific), and frozen OCT samples were sectioned at 9 μm using a Leica CM 3050S cryostat (Leica Microsystems Nussloch GmbH, Germany) and mounted on glass slides.

**TABLE 1:** Number of injected animals and collected samples per group. Aliquots (2 μL) of AAV2 (1 × 10^12^ vg/mL), AAV9 (5 × 10^12^ vg/mL), and AAV9-empty (6 × 10^11^ vg/mL) viral vectors were injected into both eyes of mice either subretinally or intravitreally. Organs were collected for qPCR and histology for GFP expression, HE-staining, and immunohistochemistry. In addition, ERG was used to study retinal function, and naïve animals were used as a control.

	Intravitreal injection			Subretinal injection		
	AAV2-EGFP	AAV9-EGFP	AAV9-empty	AAV2-EGFP	AAV9-EGFP	AAV9-empty
Total injected	5	6	4	6	5	5
qPCR						
Eyes	3	3	3	3	3	3
Brain	3	3	3	3	3	3
Lungs	3	3	3	3	3	3
Heart	3	3	3	3	3	3
Liver	3	3	3	3	3	3
Spleen	3	3	3	3	3	3
Kidney	3	3	3	3	3	3
Testicle	3	3	3	3	3	3
Lymph node	3	3	3	3	3	3
Quadricep muscle	3	3	3	3	3	3
Histology						
Eyes	5	6	3	6	5	3
Brains	3	3	2	3	3	2
Lungs	5	6	2	6	5	2
Heart	5	6	2	6	5	2
Liver	5	6	2	6	5	2
Spleen	5	6	2	6	5	2
Kidney	5	6	2	6	5	2
Testicle	3	3	2	3	3	2
Quadricep muscle	3	3	2	3	3	2
ERG	3	3	—	3	3	—

### 2.3 Electroretinography

Retinal function was assessed by electroretinography (ERG). Animals were dark-adapted overnight, and preparations for the recording were conducted in the dark under dim red light. Animals were anesthetized as previously described, and eyes were dilated with tropicamide and hydrated with carbomer eye gel. Animals were placed on a heating pad on a platform to maintain a body temperature of approximately 38°C, inside a full-field ERG dome. A platinum needle reference electrode was placed subcutaneously on the forehead, and a platinum needle ground electrode was placed under the skin of the hind limb. The ERG was recorded using a silver loop electrode placed corneally on each eye.

ColorDome Ganzfeld full-field ERG was used to record the ERG (Espion ERG; Diagnosys LLC, Cambridge, UK). First, scotopic recordings were conducted in the dark-adapted conditions, after which the eyes were light-adapted and recorded under a photopic protocol. All signals were amplified with a band-pass setting of 1–300 Hz for scotopic and 0.3–500 Hz for photopic with a sampling frequency of 2 kHz. In the scotopic protocol, both eyes were stimulated equally with five distinct intensities of blue light: 0.003, 0.007, 0.03, 0.5, and 1 Cd × s/m^2^. Fifteen sweeps of 250 ms at each intensity were recorded with a delay of 10 s between each sweep. A 60s light-adaptation period in 20 Cd/m^2^ white light (6500 K) was applied before determining the photopic responses. The photopic protocol consisted of light-adapted responses to five distinct stimulus intensities of white light: 1, 3, 5, 10, and 20 Cd × s/m^2^ in the presence of continuous background illumination of 20 Cd/m^2^. Twenty-five sweeps of 300 ms were recorded with a delay of 5 s between each sweep. An additional 60 s of wait time was included between the changes in stimulus intensities. The baseline was set identically for all recording protocols using the average voltage reading from the duration of 20 ms preceding the stimulus onset. The responses were then exported for off-site analysis.

### 2.4 ERG signal processing and feature extraction

Post-processing of the exported ERG data was performed with MATLAB (MathWorks^®^ MATLAB^®^ R2018b). For a-wave analysis, the sweeps were averaged, and the a-wave trough was determined as the lowest point of the signal, following stimulus onset and preceding the rising phase of the b-wave. The individual sweeps were then low-pass filtered using a fifth-order Bessel filter with a stopband edge frequency of 60 Hz and averaged for each stimulus intensity or frequency. The b-waves were mapped on the averaged Bessel-filtered signal by fitting a second-order polynomial in the surrounding of the highest value of voltage following an a-wave. The width of the polynomial was dynamically set as twice the time elapsed from the a-wave trough to the highest voltage point of the signal (initial estimate of the b-wave peak). The polynomial fit was iterated five times or until no change in fit parameters occurred, always in the surrounding of the peak of the parabola. All peak fits were visually checked and adjusted manually if determined inaccurate. The b-wave amplitude values were reported as the difference between the determined b-wave peak and the a-wave trough. The peak time of each wave was the time elapsed from the stimulus onset until the determined trough (a-wave) or peak (b-wave) of the signal.

The statistical analysis and plotting of the ERG results were performed with R software (version 3.5.3). The regular two-way analysis of variance (ANOVA) was used to infer statistical significance for each stimulus intensity with Bonferroni correction. The main effect between each experimental group was determined by two-way ANOVA using the injection type and stimulus intensity as independent variables. If significance was determined, the test was followed by the Bonferroni *post hoc* test with multiple pair-wise comparisons using Student’s t-test. Values of *p* < 0.05 were considered significant. Data are presented as mean ± standard deviation (SD).

### 2.5 Histology and imaging

GFP expression in the cryosections was detected using a Nikon Eclipse Ni microscope and a Nikon DS-Qi2 camera excited with a 488 nm (Tokyo, Japan) or an Olympus slide scanner (VS200 Olympus, Tokyo, Japan). A mounting medium with nuclear counterstain 4′,6-diamidino-2-phenylindole (DAPI) (H-1200, Vector Laboratories, Burlingame, CA, USA) was used. The GFP area percentage of the retinal area was determined by using Image J. The retina area was determined by drawing the retina manually, and the GFP area was determined by using threshold color and equal brightness (45/255) for all samples. The percentage of the GFP-positive area of the retinal area was calculated.

GFP transduction efficacy was verified from paraffin sections by using immunohistochemical staining for GFP [GFP (D5.1) Rabbit mAb, Cell Signaling Technology, 1:200]. Biotinylated IgG secondary antibody (Goat anti-rabbit IgG, Ba-1000 Vector Laboratories, Burlingame, CA, 1:200) was used followed by the avidin–biotin–HRP step (VECTASTAIN^®^ Elite^®^ABC Kit, Peroxidase (Standard), Pk-6100, Vector Laboratories) and DAB (DAB Substrate Kit, Peroxidase (With Nickel), SK-4100, Vector Laboratories) as a chromogen. Sections were photomicrographed with a Nikon Eclipse Ni microscope and Nikon DS-Ri2 camera or with an Olympus slide scanner (VS200, Olympus).

In addition, hematoxylin and eosin (HE) staining was used to assess the morphology of the tissue samples.

### 2.6 Quantitative PCR

DNA for quantitative polymerase chain reaction (qPCR) analysis was extracted using a NucleoSpin DNA RapidLyse kit (Macherey-Nagel) according to the manufacturer’s instructions. qPCR amplification was carried out on 50 ng of genomic DNA in duplicates. qPCR was performed using TaqMan-based assays and 2x Universal PCR Master Mix (TaqMan™, Life Technologies). Vector copy numbers were determined (StepOnePlus™ Real-Time PCR system, Applied Biosystems) using a Prime PCR assay (Bio-Rad) for an element in the vector, the woodchuck hepatitis virus post-transcriptional control element (WPRE; forward primer 5′-ATA​CGC​TGC​TTT​AAT​GCC​TTT​G-3′, reverse primer 5′-GGG​CCA​CAA​CTC​CTC​ATA​AA-3′, and probe 5′6-FAM/TCATGCTATTGCTTCCCGTATGGCT/IBFQ/-3′). Dilutions of pSub-CMV-EGFP-WPRE plasmid in duplicates were used as a standard curve. The results were calculated as mean WPRE copy numbers per microgram of genomic DNA.

## 3 Results

### 3.1 AAV9-EGFP intraocular injection transduced the retina more efficiently than AAV2-EGFP injections

**FIGURE 1:**
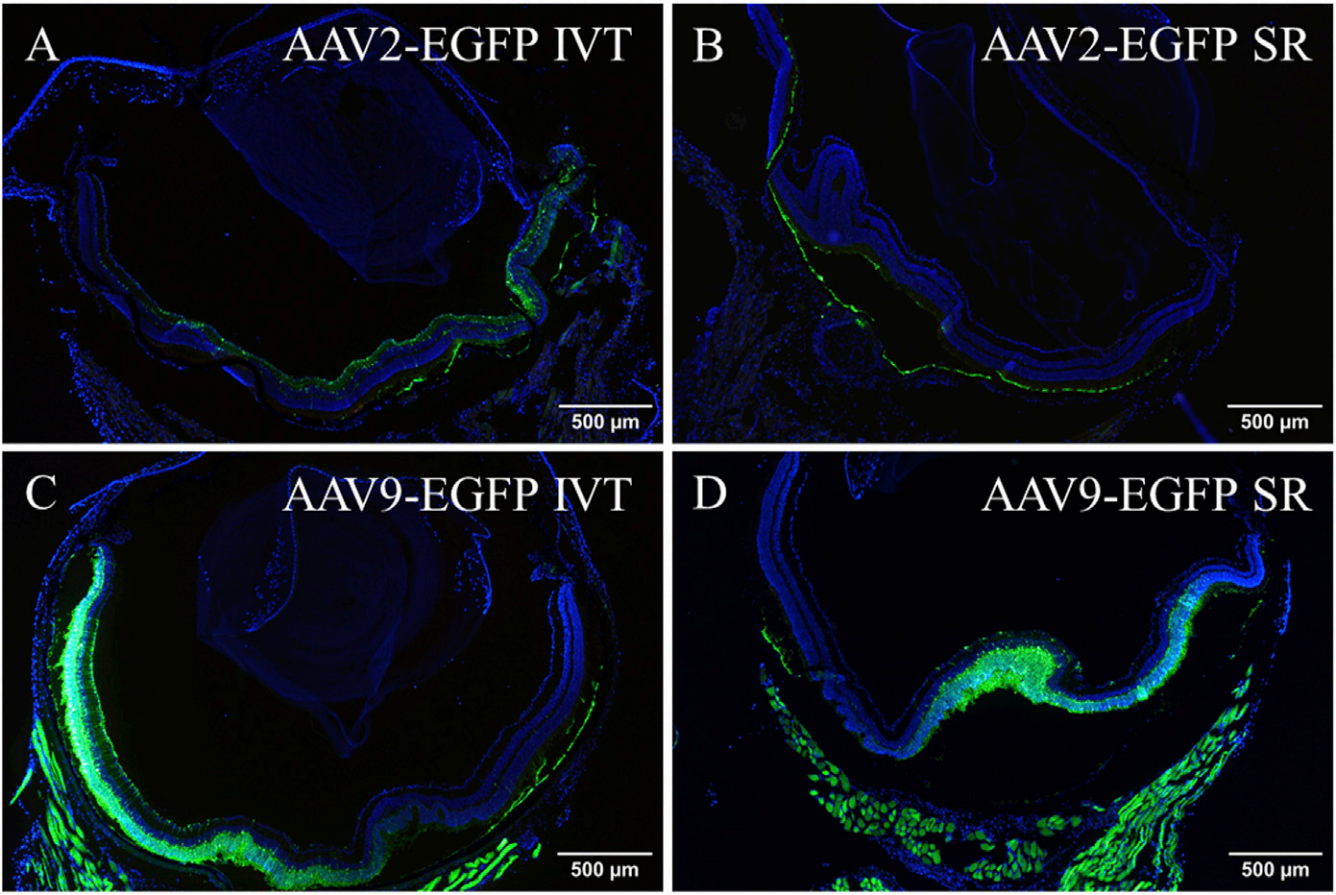
Fluorescence images showing EGFP in the eye after 1 month of AAV2-EGFP (1 × 10^12^ vg/mL) IVT **(A)** and subretinal injection **(B)**, and after AAV9-EGFP (5 × 10^12^ vg/mL) IVT **(C)** and subretinal injection **(D)**. GFP, green; Dapi, blue; IVT, intravitreal injection; SR, subretinal injection.

Transduction efficacy was evaluated from cryosectioned tissue samples. In the eye, AAV2-EGFP IVT injection transduced mainly the ganglion cell layer (GCL) and, to some extent, the inner parts of the retina and the retinal pigment epithelium (RPE) (Figure 1A). In addition, GFP was expressed in the surrounding muscles, the ciliary body (CB), and the trabecular meshwork (TM). Subretinal injection of AAV2-EGFP transduced mainly the RPE (Figure 1B), but the expression was also detected in the GCL and inner parts of the retina and surrounding muscles. After AAV9-EGFP IVT injection, GFP was expressed throughout the inner nuclear layer to the GCL and in surrounding muscles, and to some extent, in the outer nuclear layer, RPE, CB, TM, and stroma (Figure 1C). Subretinal injection of AAV9-EGFP transduced the retina throughout, mostly the outer nuclear layer and surrounding muscles and to some extent, the RPE (Figure 1D). The efficiency of AAV2-EGFP and AAV9-EGFP to transduce the retina after IVT or subretinal injections was determined using Image J. AAV9-EGFP IVT and subretinal injections were able to transduce approximately 30%–40% of the retina; AAV2-EGFP IVT injection transduced approximately 10%, and AAV2-EGFP subretinal injection transduced approximately 2% (Supplementary Figure S1).

### 3.2 EGFP expression in off-target tissues

**FIGURE 2:**
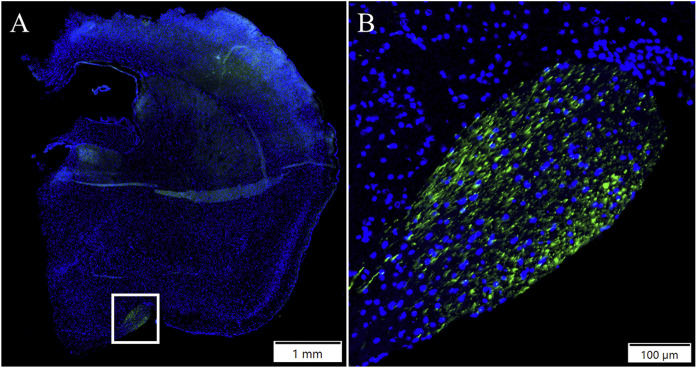
Representative image of EGFP expression in the brain **(A)** 1 month after AAV2-EGFP (1 × 10^12^ vg/mL) IVT injection. The white box indicates the GFP-positive area. ×10 magnification of the GFP positive area **(B)**. GFP, green; DAPI, blue.

AAV2- and AAV9-EGFP IVT and subretinal injection transduced a part of the brain. The expression was detected in a small area of an inferior part of the right hemisphere. Figure 2 represents EGFP expression in the brain after AAV2-EGFP IVT injection (Figures 2A, B). No expression was observed after AAV9-empty injections.

**FIGURE 3:**
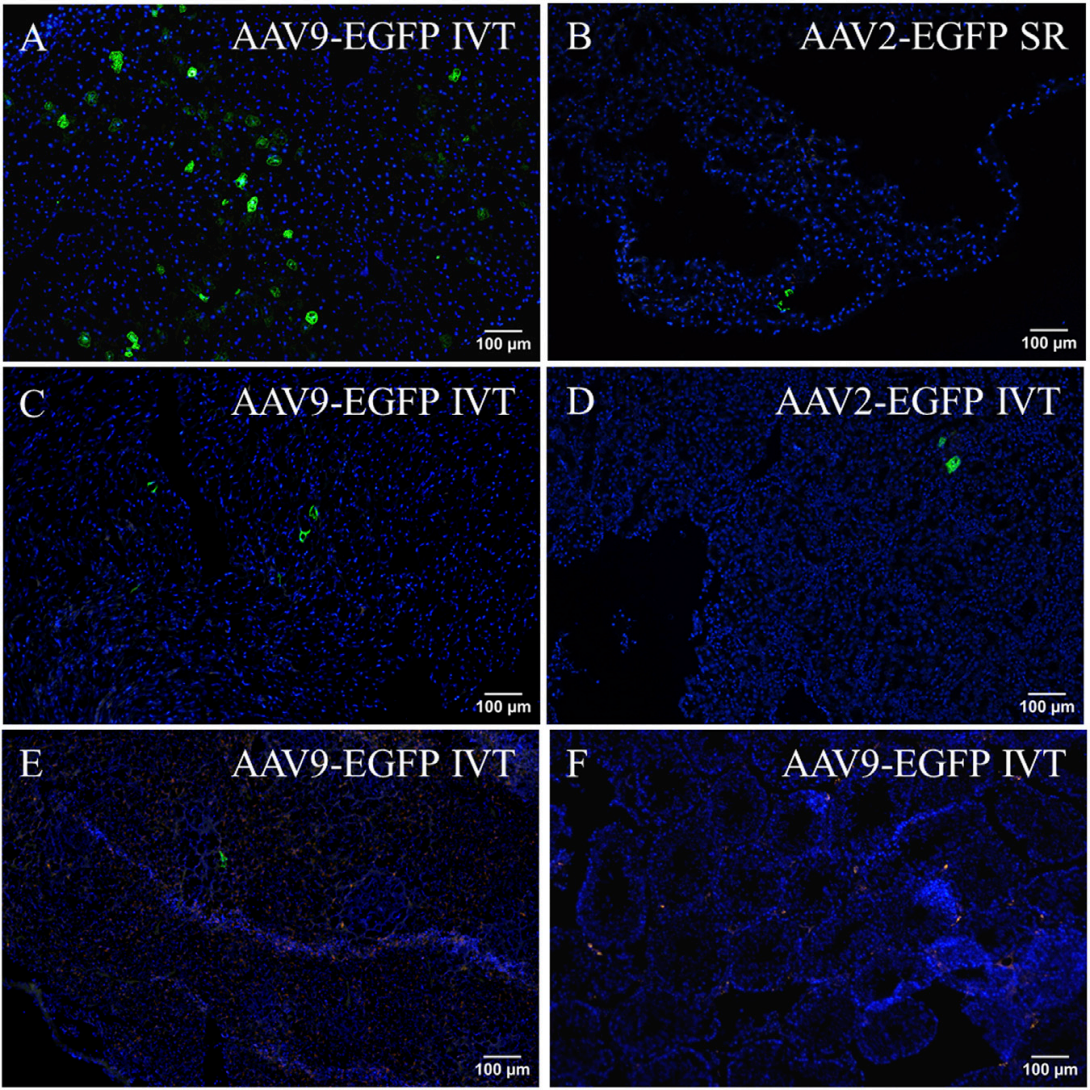
Representative images of EGFP expression in off-target tissues 1 month after AAV2-EGFP (1 × 10^12^ vg/mL) and AAV9-EGFP (5 × 10^12^ vg/mL) intraocular injections. EGFP expression in the liver after AAV9-EGFP IVT **(A)** and AAV2-EGFP subretinal injection **(B)**, the heart after AAV9-EGFP IVT injection **(C)**, the kidney after AAV2-EGFP IVT injection **(D)**, the spleen after AAV9-EGFP IVT injection **(E),** and the testicle after AAV9-EGFP IVT injection **(F)**. GFP, green; Dapi, blue; IVT, intravitreal injection; SR, subretinal injection.

Both injection routes of AAV9 transduced the liver at a moderate level (Figure 3A). In addition, AAV2-EGFP subretinal injection transduced the liver at a very low level (Figure 3B), but no expression was seen in the IVT AAV2-EGFP group. In the heart, EGFP was expressed at a low level in both of the AAV9 injection groups (Figure 3C), whereas no expression was seen in the AAV2 injection groups. In the kidney, EGFP was expressed at a low level after both AAV2- and AAV9-EGFP IVT (Figure 3D), but no expression was detected after the subretinal injection of either vector. The spleen was transduced at a very low level after both AAV9-EGFP injection routes (Figure 3E), whereas no transduction was detected in the AAV2 groups. We also determined whether gonads (testicles) were transduced after intraocular injections of AAV2-EGFP–and AAV9-EGFP, but no EGFP expression was observed (Figure 3F). The numbers of tissue samples where EGFP expression was present are presented in Table 2.

**TABLE 2:** Number of tissue samples expressing EGFP 1 month after IVT and subretinal injection of AAV2-EGFP, AAV9-EGFP, and AAV9-empty. AAV9-empty comprised two samples of IVT injection and two samples of subretinal injection.

	AAV2-EGFP		AAV9-EGFP		AAV9-empty
	IVT	Subretinal	IVT	Subretinal	
Brain	2/3	2/3	2/3	3/3	0/4
Liver	0/2	2/3	3/3	1/2	
Heart	0/2	0/3	3/3	1/2	
Lungs	0/2	0/3	0/3	0/2	
Spleen	0/2	0/3	2/3	2/2	
Kidney	0/2	0/3	3/3	0/2	
Testicle	0/2	0/3	0/3	0/3	0/4

### 3.3 GFP immunohistostaining

**FIGURE 4:**
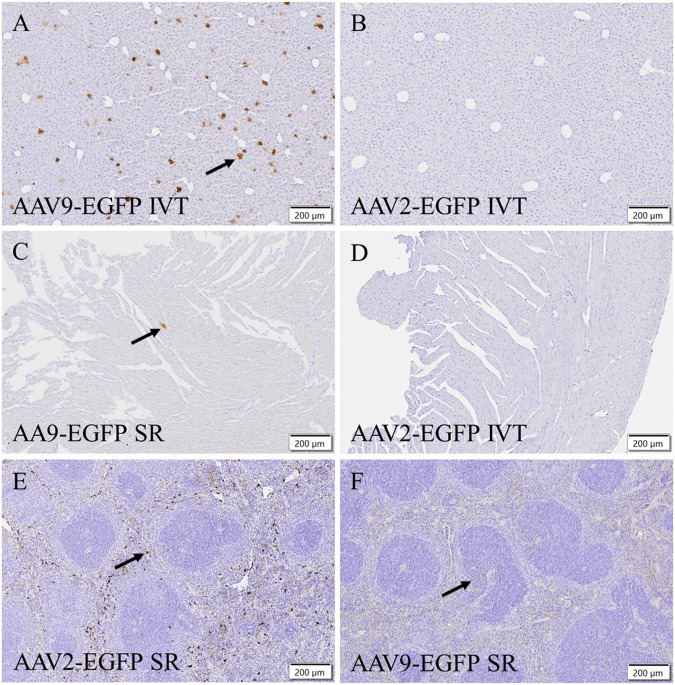
Representative images of EGFP immunostaining of positive cells (black arrow) after the IVT or subretinal injection of AAV9-EGFP (5 × 10^12^ vg/mL) and AAV2-EGFP (1 × 10^12^ vg/mL). GFP in the liver after AAV9-EGFP IVT injection **(A)** and after AAV2-EGFP IVT injection **(B)**. In the heart after AAV9-EGFP subretinal injection **(C)** and after AAV2-EGFP IVT injection **(D)**. In the spleen after subretinal injection of AAV2-EGFP **(E)** and AAV9-EGFP **(F)**. IVT, intravitreal injection; SR, subretinal injection.

To verify EGFP transduction efficacy, immunostaining for GFP was performed. Subretinal injection of AAV9-EGFP transduced the liver at a moderate level (Figure 4A). A similar level of transduction was seen in the AAV9-EGFP-expressing IVT group. No transduction was seen after AAV2-EGFP IVT (Figure 4B) and subretinal injection. In the heart, some positive cells were observed after AAV9-EGFP subretinal injection (Figure 4C) in one of three samples and in two of three samples after AAV9-EGFP IVT injection. In addition, a few positive cells were observed in one heart sample after AAV2-EGFP subretinal injection, but no transduction was seen after AAV2-EGFP IVT injection (Figure 4D). GFP-positive cells were observed in two of three samples of the spleen after AAV2-EGFP subretinal injection (Figure 4E), and after AAV2-EGFP IVT injection in one of three samples at a moderate level. The spleen was also transduced at a low level after AAV9-EGFP IVT and subretinal injection (Figure 4F). No GFP-positive cells were observed in any tissue after AAV9-empty injections.

**FIGURE 5:**
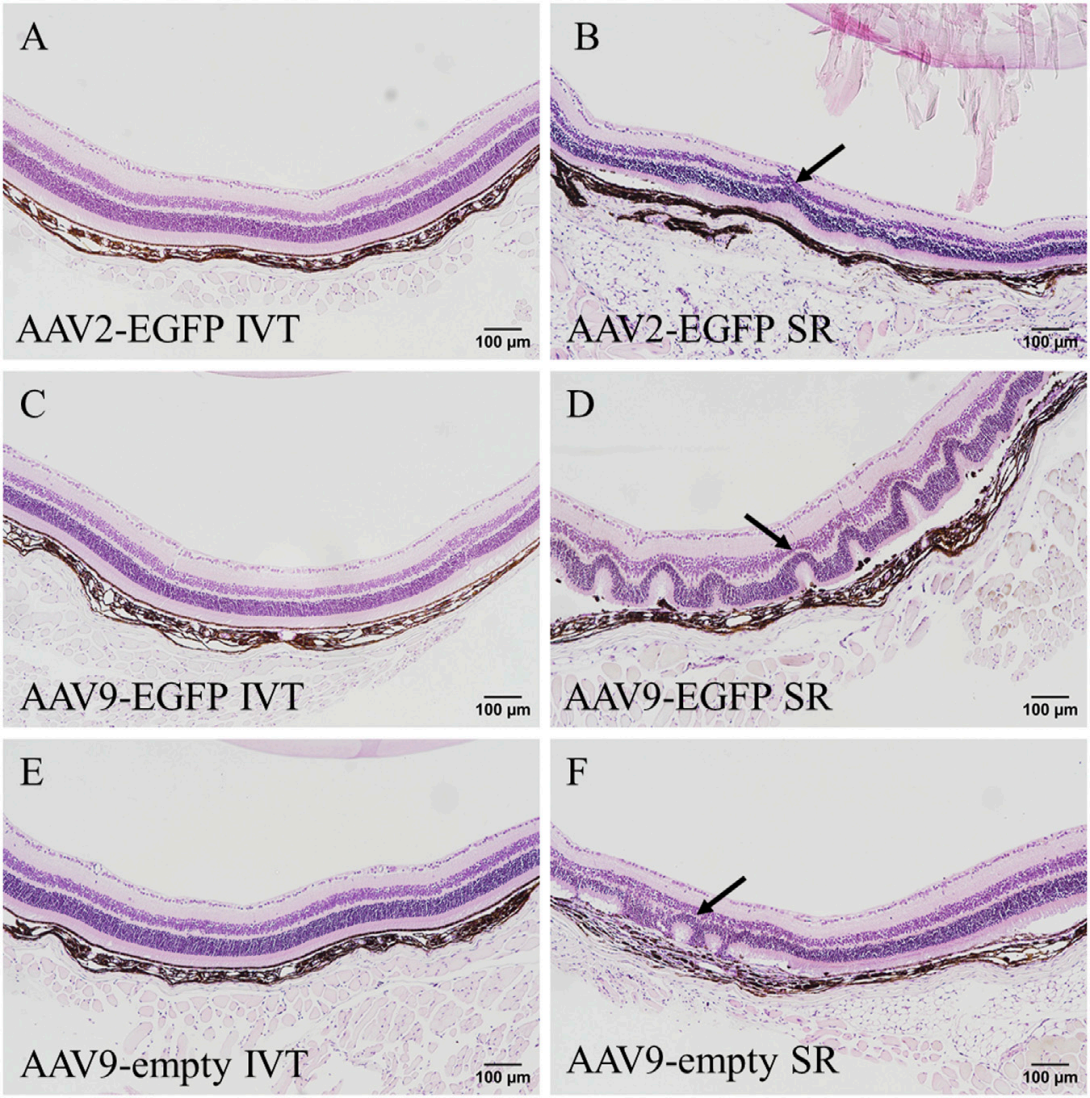
HE-stained eye sections after 1 month of intraocular injections of AAV2-EGFP (1 × 10^12^ vg/mL) **(A,B)**, AAV9-EGFP (5 × 10^12^ vg/mL) **(C,D)**, and AAV9-empty (6 × 10^11^ vg/mL) **(E,F)**; black arrow indicates abnormal morphology seen in the retina. IVT, intravitreal injection; SR, subretinal injection.

The general histology of the tissues was assessed from HE-stained sections. No abnormalities were seen in the retina after AAV2-EGFP (Figure 5A), AAV9-EGFP (Figure 5C), and AAV9-empty (Figure 5E) IVT injections. After subretinal injection of AAV2-EGFP (Figure 5B), AAV9-EGFP (Figure 5D), and AAV9-empty (Figure 5F), some abnormalities in the retina morphology were observed. Especially, AAV9-EGFP subretinal injection caused retinal detachment and retinal folding. No abnormalities were seen in any other stained tissue sample.

### 3.4 qPCR

**FIGURE 6:**
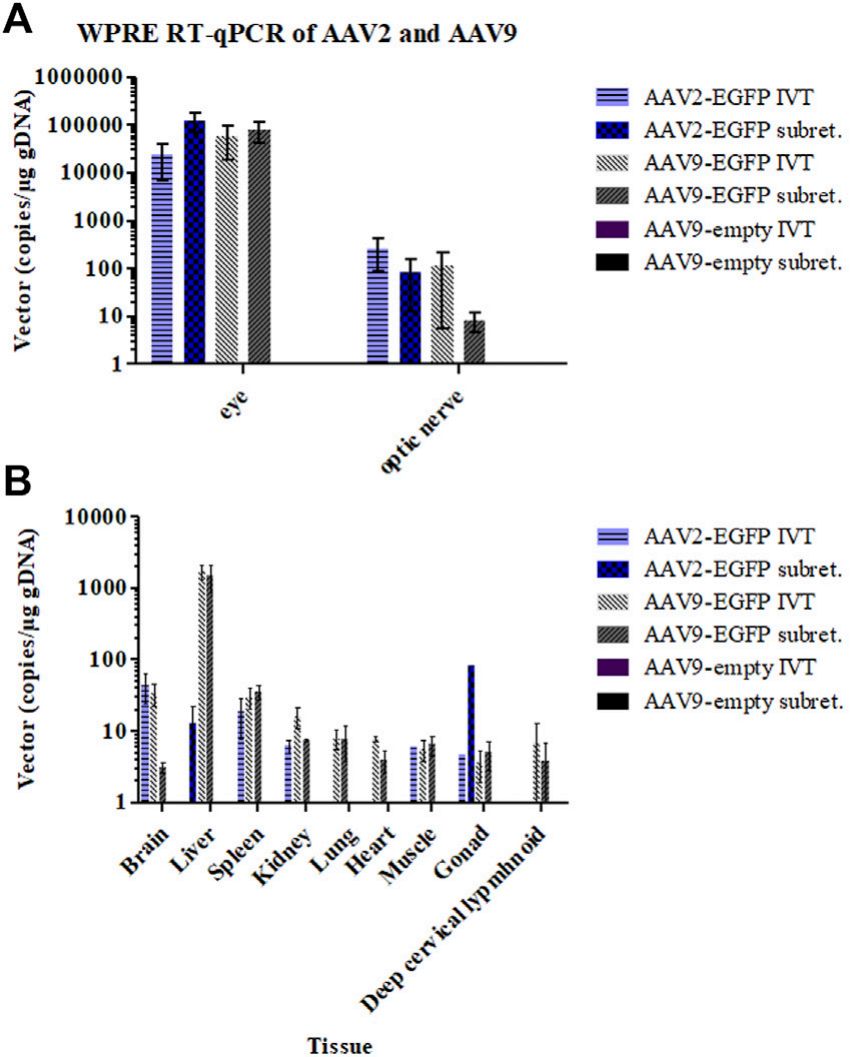
Viral vector copy numbers per total genomic DNA in the eye and optic nerve samples 1 month after the IVT and subretinal injections of AAV2- and AAV9-EGFP **(A)** and in other tissues **(B)**.

Genomic DNA was extracted, and qPCR was performed to assess the biodistribution in the major tissues after IVT or subretinal injections of AAV2- and AAV9-EGFP vectors. The mean WPRE copy numbers/microgram of genomic DNA was calculated. AAV9-empty did not contain WPRE. Viral vectors were found mostly in the eye and the optic nerve in all of the groups except AAV9-empty (Figure 6A). Vector copies were also found in all collected tissues (the brain, spleen, kidney, lung, heart, quadriceps muscle, gonads, and deep cervical lymphoid) and especially in the liver after IVT and subretinal injections of AAV9-EGFP (Figure 6B). Low levels of vector copies were found in the brain, spleen, kidney, quadriceps muscle, and gonads after AAV2-EGFP IVT. After subretinal injection, AAV2-EGFP vector copies were found at low levels in the liver and one gonad sample. No vector copies were found in the brain, heart, lung, liver, kidney, or spleen. There was also less than one copy/µg of genomic DNA in the deep cervical lymph nodes after both injection routes of AAV2-EGFP. No vector copies were found in any of the tissues after AAV9-empty IVT or subretinal injection.

### 3.5 Electroretinography results

**FIGURE 7:**
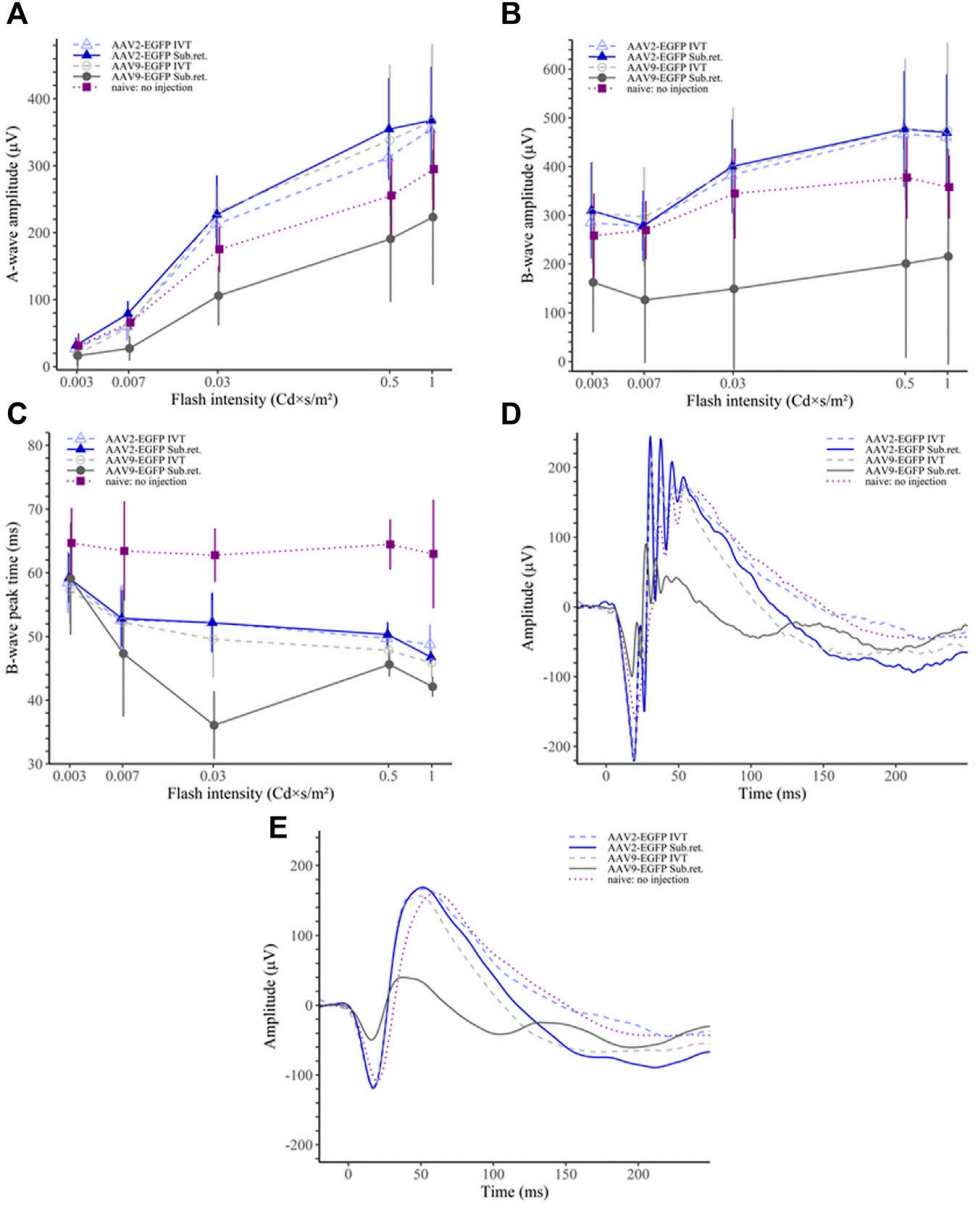
Scotopic ERG results. **(A)** The AAV9 subretinal group had significantly reduced ERG a-wave amplitudes than any other group. **(B)** Similarly, the b-wave amplitudes of the AAV9 subretinal group were significantly reduced in comparison to other groups. **(C)** The b-wave peak time was the shortest among the AAV9 subretinal group. On the contrary, the no-injection group had significantly prolonged peak time as compared to any other group. **(D)** Averaged raw ERG signals of each group at a flash intensity of 0.03 Cd × s/m^2^. **(E)** Filtered (5th order low-pass Bessel filter with a cut-off at 60 Hz) ERG signals of the same responses as in **(D)**.

In the scotopic ERG, the AAV9 subretinal group had significantly reduced a-wave amplitudes compared to any other group (*p* < 0.05 for naive, *p* < 0.01 for the rest; (Figure 7A). Similarly, the b-wave amplitudes were significantly downregulated in the AAV9 subretinal group (*p* < 0.05 for naive, *p* < 0.01 for the rest; Figure 7B). Interestingly, the b-wave peak time was observed to shorten in the AAV9 subretinal group (*p* < 0.01 for naive and AAV2 subretinal and IVT groups; Figure 7C), while the peak time was significantly prolonged in the naive group (*p* < 0.001 for each group). The averaged raw responses for each group are visualized at the stimulus intensity of 0.03 Cd × s/m^2^ in Figure 7D and the filtered responses at the same intensity in Figure 7E.

**FIGURE 8:**
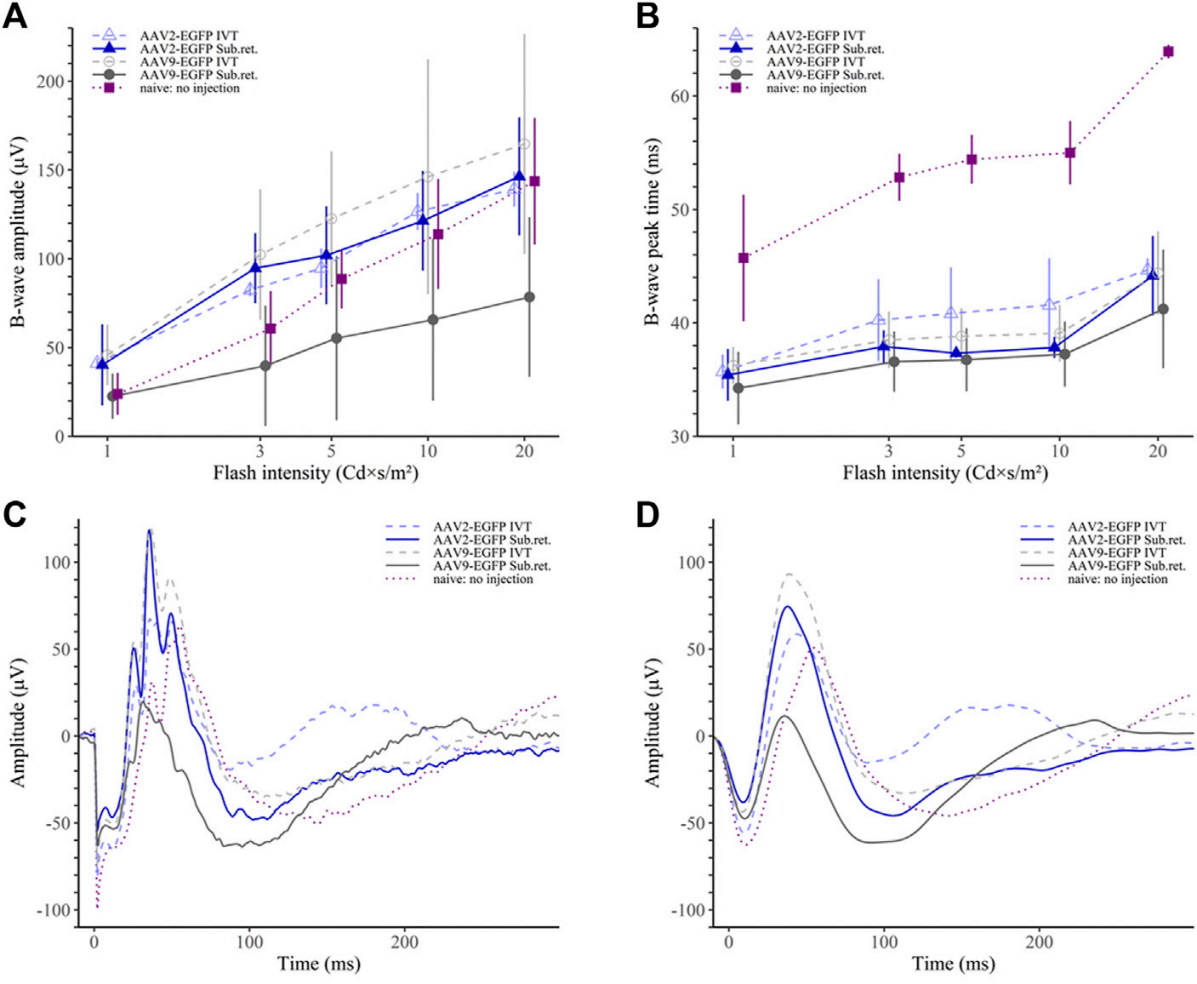
Photopic ERG results. **(A)** The AAV9 subretinal group had statistically lower b-wave amplitudes than the AAV9 IVT and the AAV2 IVT groups. **(B)** The b-wave peak time was the shortest in the AAV9 subretinal group. On the contrary, the no-injection group had a significantly prolonged peak time in comparison to any other group. **(C)** Averaged raw ERG signals of each group at a flash intensity of 10 Cd × s/m^2^. **(D)** Filtered (5th order low-pass Bessel filter with a cut-off at 60 Hz) ERG signals of the same responses as in **(C)**.

In the photopic ERG, the AAV9 subretinal group had significantly reduced b-wave amplitude only compared to the AAV9 IVT group (*p* < 0.05; Figure 8A). Similarly, as in the scotopic ERG, the b-wave peak time was observed to be shorter in the AAV9 subretinal group compared to any other group (*p* < 0.001 for naive, *p* < 0.01 for AAV2 IVT and AAV9 IVT, *p* < 0.05 for AAV2 subretinal; Figure 8B). The peak time was significantly prolonged in the naive group (*p* < 0.001 in comparison to any other group). The averaged raw responses for each group are visualized at the stimulus intensity of 10 Cd × s/m^2^ in Figure 8C and the filtered responses at the same intensity in Figure 8D.

## 4 Discussion

In this study, we assessed the efficacy, biodistribution, and safety of AAV2-EGFP and AAV9-EGFP IVT and subretinal injections. Intraocular injections of AAV9-EGFP transduced the retina (30%–40%) more efficiently than AAV2-EGFP (2%–10%) as detected from cryosections. IVT injection potentially exposes the entire retina to gene therapy (Koponen et al., 2021; Puranen et al., 2022), whereas delivery to subretinal space allows direct contact of the vector with photoreceptors and the RPE layers (Irigoyen et al., 2022). The AAV2-EGFP IVT injection transduced mainly GCL and subretinal injection RPE, whereas GFP expression was detected throughout the retina after both AAV9-EGFP injection routes. In previous studies, AAV2 has been shown to transduce the GCL, inner parts of the retina, and the optic nerve after IVT injection in mice (Hellström et al., 2009; Kalesnykas et al., 2017) and the RPE and photoreceptors after subretinal injection (Pang et al., 2008). AAV9 transduction has been detected throughout the retina after subretinal injections (Lei et al., 2009; Han et al., 2020) but with limited transduction efficacy with IVT injection (Lee et al., 2018b), although, in this study, IVT injection of AAV9-EGFP transduced the retina efficiently. In addition, intravenous injection of AAV9-GFP via the tail vein has been shown to induce retinal (RPE, PR, ONL, OPL, INL, IPL, and GCL), choroidal, and optic nerve transduction in mice, indicating that AAV9 is able to cross the blood–retinal barrier (Bemelmans et al., 2013). AAV9-EGFP subretinal injection caused more morphological changes, such as retinal detachment and folding, than any other group. Some changes were also detected after the AAV9-empty and, at a lower level, after the AAV2-EGFP subretinal injection. Previous studies have reported morphological changes in the retina related to subretinal injection (Fisher et al., 2005; Fernandez-Bueno et al., 2019), vector dose (Jacobson et al., 2006; Beltran et al., 2010; Xiong et al., 2019), promoter used (Beltran et al., 2010; Xiong et al., 2019), or the immunogenicity and toxicity of the GFP (Khabou et al., 2018).

Several factors can affect the transduction efficiency of AAVs in the eye, such as ocular barriers, delivery route, immune responses, vector design, AAV serotype, and vector dose. In disease models, such as streptozotocin (STZ)-induced diabetic mice (Lee et al., 2018b) and the laser-induced choroidal neovascularization mouse model (Lee et al., 2018a), AAV-mediated transduction has been shown to be enhanced with certain AAV serotypes. Barriers like the inner limiting membrane (ILM) limit the efficient gene delivery to the retina when the vector is administered intravitreally (Dalkara et al., 2009). Rodents have a thinner ILM than humans and larger animals, such as dogs and monkeys, which must be considered when studying the efficacy of gene delivery into the retina. One solution to enhancing AAV-mediated transduction efficiency could be removing the ILM enzymatically (Dalkara et al., 2009), surgically (Teo et al., 2018), or by injecting between the ILM and neural retina (Boye et al., 2016). Capsid modifications have been shown to enhance transgene delivery to the outer retina after IVT (Petrs-Silva et al., 2009; Dalkara et al., 2013; Katada et al., 2019; Pavlou et al., 2021). Promoter optimization has been shown to transduce targeted cells efficiently (Beltran et al., 2017; Hanlon et al., 2017) and also has been shown to limit unwanted off-target cell transduction (Lu et al., 2016; Beltran et al., 2017; Hanlon et al., 2017). In addition, higher vector doses have been shown to enhance transduction efficiency (Vandenberghe et al., 2011). However, a higher vector dose can elicit immune responses (Barker et al., 2009; Cukras et al., 2018). This study used a dose of 7 × 10^10^ vg/kg/eye. In clinical trials, doses in the eye of 1.5E10 vg in 150 mL (NCT00516477, 2020), 1.5 E11 vg in 0.3 mL (NCT00999609, 2023) or 1.1 × 10E12 vg (NCT03585556, 2022) have been used.

AAV2 has a tropism for smooth muscle, skeletal muscle, central nervous system, liver, and kidney, and AAV9 has a tropism for the liver, heart, brain, skeletal muscle, lungs, pancreas, and kidney (Verdera et al., 2020). Differences in the tropism are determined by the AAV capsid, the primary attachment receptor, and the co-receptor specificity of each serotype (Colón-Thillet et al., 2021). In this study, after both injection routes, AAV9-EGFP vector copies were found in all tissues analyzed, especially in the liver. Low levels of AAV2-EGFP vector copies were found in the brain, kidney, and spleen after IVT and in the liver after subretinal injection. In addition, vector copies were found in one gonad sample after both AAV2 injection routes. Transgene expression was observed in the liver, heart, and spleen after both AAV9-EGFP injection routes and in the kidney after AAV9-EGFP IVT. However, fewer tissue samples expressed EGFP in the subretinal group than in the IVT group. The subretinal space is thought to be anatomically more closed and immune-privileged than the intravitreal space (Irigoyen et al., 2022). Drugs are eliminated from the vitreous cavity *via* blood–ocular barriers and aqueous humor outflow to the systemic blood circulation (del Amo et al., 2017). A previous study showed that IVT injection of AAV8 leads to more persistent systemic exposure than subretinal injection (Seitz et al., 2017). After AAV2-EGFP IVT injection, expression was observed in the spleen, and after AAV2-EGFP subretinal injection, expression was observed in the liver. Studies have shown low levels of vector copies in distal organs, including the liver, brain, and spleen, after IVT of AAV2 (MacLachlan et al., 2011) and in the liver, spleen, kidney, muscle, lung, and heart after intravenous injection (Mori et al., 2004). Vector copies have been shown to be present in the liver, lung, heart, kidney, testes, brain, and muscle after intravenous injection of AAV9 (Zincarelli et al., 2008). Our data are consistent with these studies.

In previous studies, vector sequences have been detected in the brain and optic nerve after AAV2 IVT injection in rats, dogs (Provost et al., 2005), mice (Griffey et al., 2005), and nonhuman primates (MacLachlan et al., 2011; Calkins et al., 2021). Vector copies in the brain have been found, especially in the regions that constitute the visual pathway in dogs (Provost et al., 2005) and mice (Griffey et al., 2005). It has been reported that AAV2 can undergo anterograde axonal transport in rat and primate brains (Salegio et al., 2013). In addition, a previous study showed transgene expression in the optic nerve and brain after AAV2-GFP IVT injection (Dudus et al., 1999). We also found GFP expression in brain samples after both injection routes of AAV2-EGFP and AAV9-EGFP. In a previous study, vector copies were found in the optic nerve in rats but not in the brain after subretinal injection of AAV2 (Provost et al., 2005), whereas no vector copies were found in the optic nerve or brain in dogs except in one of 22 dogs (Jacobson et al., 2006).

ERG was used to infer the safety of IVT and subretinal injection of AAV vectors. ERG evaluates the retinal function as an electrical potential difference between the retina and cornea, consisting of a sum response of various retinal cell layers (Perlman, 1995). In our study, the AAV9-EGFP subretinal group consistently showed lowered a- and b-wave amplitude values in both scotopic and photopic experiments. This could implicate both rod- and cone-focused pathway dependency being compromised in this group because the a-wave originates from the photoreceptor level, and the b-wave is mainly composed of bipolar cell response (Brown, 1968; Stockton and Slaughter, 1989; Pugh et al., 1998). In addition, morphological changes were detected after the subretinal injection of AAV9-EGFP. In contrast, both of the AAV2 groups sustained a similar retinal function to that AAV9-EGFP IVT group in the experiments. In a previous study, AAV9 subretinal injection has been shown to lower the amplitude of oscillatory potentials in dark-adapted b-waves compared to the untreated eye (Lei et al., 2009). However, a similar reduction was observed after the HEPES injection, suggesting that the reduction is possibly related to the injection procedure. In our study, the naive group that had no injection and acted as a control had lower b-wave amplitude values than the AAV2 groups and the AAV9 IVT group but higher b-wave amplitudes than the AAV9 subretinal group. Interestingly, the b-wave peak time was significantly higher in both scotopic and photopic ERG in the naive group than in any other group. Therefore, it would seem that any injection we administered resulted in a faster peak time occurrence. This earlier attainment of the peak could stem from the faster photoreceptor depolarization and transmittance to bipolar cells. However, because the a-wave peak times between the naive and other groups were not significantly different, the earlier attainment of the b-wave peak could stem from faster activation of the bipolar cells and have little to do with the photoreceptor level. This could be, for instance, due to enhanced propagation from photoreceptors to bipolar cells.

Gene therapy products, such as Luxturna (voretigene neparvovec), used to treat inherited biallelic RPE65 mutation-associated retinal dystrophy, Zolgensma (onasemnogene abeparvovec), used to treat spinal muscular atrophy and approved by the EMA (EMA, 2018; EMA, 2020) and the FDA (FDA, 2022), Roctavian (valoctocogene roxaparvovec), used to treat hemophilia A, Upstaza (eladocagene exuparvovec), used to treat aromatic L-amino acid decarboxylase (AADC) deficiency and approved by the EMA (EMA, 2022a; EMA, 2022b), and Adstiladrin (nadofaragene firadenovec-vncg), used to treat *Bacillus* Calmette-Guérin (BCG)-unresponsive nonmuscle invasive bladder cancer and approved by the FDA (FDA, 2022), prove that gene therapy is a useful tool in the treatment of various diseases. Of these five gene therapies, two are AAV2-mediated, and one is mediated by AAV9. AAV2 is being widely studied in clinics and is being used in gene therapies with marketing authorization, whereas AAV9 is not currently used in any clinical trials treating ocular-related diseases (Zhao et al., 2022). However, AAV9 has been shown to be efficient in animal studies (Vandenberghe et al., 2013; Lee et al., 2018b; Ail et al., 2022).

In conclusion, IVT and subretinal injections of AAV9-EGFP were able to transduce the retina more efficiently than AAV2-EGFP injections. The transduction pattern in the eye is determined largely by the injection route; the inner parts of the retina are transduced by IVT injection, while subretinal injection mainly transduces the outer parts of the retina and the RPE. Although AAV9-mediated gene delivery was more effective in transducing the retina, a negative effect on retinal function and more widespread off-target expression were observed. These results indicate that AAV2 is a more suitable gene delivery vector to treat ocular disorders.

## Data Availability

The original contributions presented in the study are included in the article/Supplementary Materials; further inquiries can be directed to the corresponding author.
