# Supplementary figures and images for: AAV2 and AAV9 tropism and transgene expression in the mouse eye and major tissues after intravitreal and subretinal delivery
Source: Front Drug Deliv. 2023 Jul 12;3:1148795. doi: 10.3389/fddev.2023.1148795 (PMC12363253; doi:10.3389/fddev.2023.1148795)

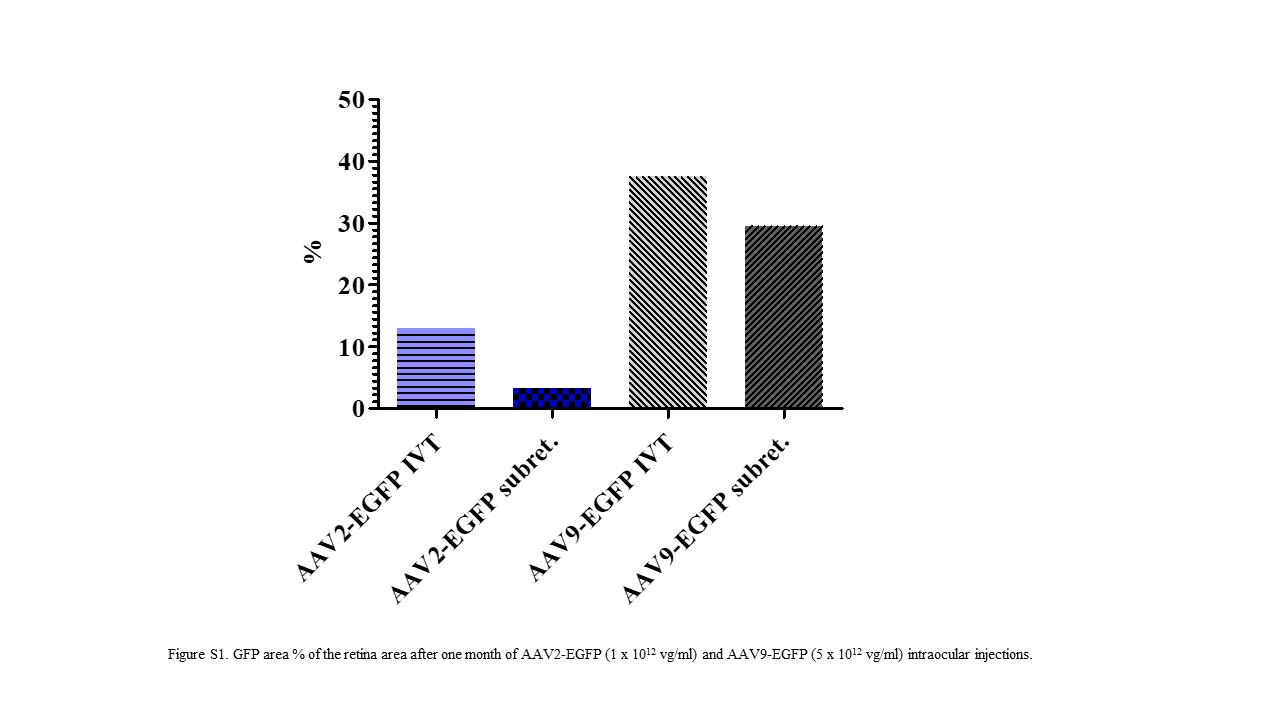

Supplement: Supplementary file 1 [file Image1.JPEG]
